# Designing and Developing Online Training for Diabetes Prevention Program Coaches Using an Integrated Knowledge Translation Approach: Development and Usability Study

**DOI:** 10.2196/50942

**Published:** 2024-01-26

**Authors:** Kaela D Cranston, Natalie J Grieve, Tineke E Dineen, Mary E Jung

**Affiliations:** 1 Faculty of Health and Social Development University of British Columbia Kelowna, BC Canada

**Keywords:** program evaluation, prediabetic state, e-learning education, e-learning, platform, usability, diabetes, prevention, knowledge translation, end user, type 2 diabetes, framework

## Abstract

**Background:**

e-Learning has rapidly become a popular alternative to in-person learning due to its flexibility, convenience, and wide reach. Using a systematic and partnered process to transfer in-person training to an e-learning platform helps to ensure the training will be effective and acceptable to learners.

**Objective:**

This study aimed to develop an e-learning platform for Small Steps for Big Changes (SSBC) type 2 diabetes prevention program coaches to improve the viability of coach training.

**Methods:**

An integrated knowledge translation approach was used in the first 3 stages of the technology-enhanced learning (TEL) evaluation framework to address the study objective. This included three steps: (1) conducting a needs analysis based on focus groups with previously trained SSBC coaches, meetings with the SSBC research team, and a review of research results on the effectiveness of the previous in-person version of the training; (2) documenting processes and decisions in the design and development of the e-learning training platform; and (3) performing usability testing. Previously trained SSBC coaches and the SSBC research team were included in all stages of this study.

**Results:**

Step 1 identified components from the in-person training that should be maintained in the e-learning training (ie, a focus on motivational interviewing), additional components to be added to the e-learning training (ie, how to deliver culturally safe and inclusive care), and mode of delivery (videos and opportunities to synchronously practice skills). Step 2 documented the processes and decisions made in the design and development of the e-learning training, including the resources (ie, time and finances) used, the content of the training modules, and how coaches would flow through the training process. The design and development process consisted of creating a blueprint of the training. The training included 7 e-learning modules, the learning modalities of which included narrated demonstration videos and user-engaging activities, a mock session with feedback from the research team, and a final knowledge test. Step 3, usability testing, demonstrated high levels of learnability, efficiency, memorability, and satisfaction, with minor bugs documented and resolved.

**Conclusions:**

Using an integrated knowledge translation approach to the technology-enhanced learning evaluation framework was successful in developing an e-learning training platform for SSBC coaches. Incorporating end users in this process can increase the chances that the e-learning training platform is usable, engaging, and acceptable. Future research will include examining the satisfaction of coaches using the SSBC coach e-learning training platform, assessing coach learning outcomes (ie, knowledge and behavior), and estimating the cost and viability of implementing this training.

## Introduction

e-Learning has emerged as a popular alternative to traditional in-person education in recent years, particularly with the advancement of technology and widespread availability of internet connectivity. e-Learning has revolutionized the way people learn by providing flexible, wide-reaching, and convenient access to educational resources, as well as personalized learning experiences that cater to individual needs and preferences. e-Learning has been adopted across various fields, including health care, and is effective in enhancing learning outcomes and improving overall learner engagement [1-3].

There is a paucity of research on how e-learning platforms are designed and developed. Partnering with end users can help guide e-learning platform development. An integrated knowledge translation (IKT) approach meaningfully engages the right research users at the right time throughout the research process and can increase the chances that the e-learning platform is usable, engaging, and acceptable [4-7]. Such approaches require in-depth qualitative methods to understand the perspectives of end users throughout the entire research process.

Frameworks can help guide the process of platform conception, design, development, implementation, and evaluation. This study was guided by the technology-enhanced learning (TEL) evaluation framework [8]. The TEL evaluation framework was informed by commonly used learning models, including the Kirkpatrick model [9]. The seven TEL evaluation framework stages are (1) conduct needs analysis and environmental scan; (2) document processes, decisions, and final product; (3) test usability; (4) document key events during implementation and final product; (5) assess participant experience and satisfaction; (6) assess learning outcomes; and (7) estimate cost, reusability, and sustainability. The TEL lacks specific guidelines, which can be seen as both a strength and a limitation, as it permits flexibility for different users and contexts, but it can also be used haphazardly and may result in a product that has not considered end user needs.

This paper offers a guide for including an IKT approach to the needs analysis, design, development, and usability testing of an e-learning platform using the first 3 stages of the TEL evaluation framework. The proposed partnered stages of the TEL evaluation framework are described using the example of developing an e-learning platform for type 2 diabetes (T2D) prevention program coaches.

## Methods

### Context

Small Steps for Big Changes (SSBC) is a community-based T2D prevention program that aims to empower individuals with prediabetes to increase physical activity and improve their diet to reduce their risk of developing T2D. SSBC is delivered in and by staff and volunteers of the YMCA of Southern Interior British Columbia. Trained SSBC coaches (YMCA staff and volunteers) deliver 6 one-on-one sessions to clients over 4 weeks using a motivational interviewing (MI)-informed approach. In-person 3-day workshops were previously used to train coaches to deliver SSBC. After completing the in-person training, coaches delivered the SSBC program with good levels of fidelity [10,11]. However, in-person coach training is not a viable format for future coach training due to difficulty in scheduling training time and the inability to revisit training components. Additionally, the in-person training was not developed using an IKT approach. To ensure usability and acceptability, the development of the SSBC coach e-learning platform used an IKT approach (ie, partnering with current SSBC coaches).

### Needs Analysis

#### Overview

The information was gathered on the program needs and capacities (eg, the specific knowledge and skills required to become an SSBC coach) and individual needs of SSBC coaches (eg, preference for specific content and instructional approach). The various components of the needs analysis stage were assessed through meetings with the SSBC research team, focus groups with previously trained SSBC coaches, and analysis of previous research.

#### Previous Research

Dineen et al [10] evaluated the effectiveness of the SSBC in-person training. Data from this study helped determine coaches’ baseline levels of knowledge and skills prior to any training.

#### SSBC Research Team Meetings

The SSBC research team included the founder and director of SSBC (the last author) and the 2 in-person training facilitators (first and third author). The first meeting was held to determine whether the in-person training was a feasible and viable mode to continue to train SSBC coaches. The main reasons discussed for transitioning to e-learning were difficulties scheduling a 3-day in-person SSBC coach training for YMCA staff and volunteers, and concerns for practicality in the future expansion of SSBC to other Canadian and global facilities. Additional meetings with the research team were used to determine what knowledge and skills would be required of new SSBC coaches, what content from the in-person training would be included and excluded, and what new concepts could be added. Finally, SSBC research team meetings included discussions on measures to determine the effectiveness of the e-learning platform. In-depth meeting notes and minutes were recorded during meetings for later analysis.

#### Focus Groups With SSBC Coaches

Incorporating end users into the development of the e-learning platform (a main tenet of IKT) was done throughout the needs analysis, design, and development of the SSBC coach e-learning platform. Within the need analysis, focus groups with previously trained SSBC coaches were conducted. SSBC coaches who were trained through the in-person workshop were recruited via email to participate in a 2-hour focus group. Focus group questions centered on what content should or should not be included in the e-learning platform. Coaches also answered questions about motivation to learn, learning preferences (ie, synchronicity, length of time, interactivity, and choice), and potential barriers for new coaches. Focus group data were analyzed using conventional content analysis [12].

### Design and Development

To facilitate the design and development phase of this project, the research team hired a third-party digital health solutions company (3C Institute) that specializes in developing e-learning platforms. The first and last author had meetings with 3C Institute every 2 weeks to discuss project progress and make decisions. Throughout this time, the research team created a blueprint and storyboard of the detailed topics to be taught, discussed options for teaching formats (ie, a variety of didactic and user-engaging methods), and defined the learning objectives. The research team wrote the content for the module scripts, which were edited by 3C Institute to ensure they were clear and succinct. Modules were filmed by the research team, and 3C Institute developed graphics and compiled the modules.

SSBC coaches from the needs analysis phase were invited to participate in the design and development phase, specifically to review the overview of modules and methods of instruction and provide feedback.

All processes and decisions were documented in detailed meeting notes (eg, attendees, discussion points, and decisions). Calendar events and contracts captured the resources (eg, financial and time) required to develop the e-learning platform.

### Test Usability

Usability evaluation helps to ensure that a product is usable, efficient, effective, and satisfying to its end users. Nielsen [13] identified five key components of usability: (1) learnability—how easily end users can accomplish basic tasks the first time they enter the e-learning platform; (2) efficiency—how well end users can perform tasks on the e-learning platform; (3) memorability—how easily end users can reestablish proficiency when they return to the e-learning platform after a period of not using it; (4) errors—how many errors the end users make, the severity of the errors, and their ease of recovery from the errors; and (5) satisfaction—how pleasant it is to use the e-learning platform.

Initial usability testing was conducted by the SSBC research team and the SSBC coaches involved in the e-learning platform design and development. Users were asked to navigate through the modules and learning activities and complete various tasks (eg, log in to the training platform, move through modules, and access the resource center). Written and verbal feedback on issues and errors were collected. A summary of concerns and usability issues was documented and sent to 3C Institute to be addressed. The level of usability was deemed acceptable by the first and last authors once all errors were resolved. In the true spirit of IKT, further usability testing will be examined through qualitative and quantitative methods upon release and implementation of the e-learning platform to ensure that the platform is usable by the end users.

### Ethical Considerations

This study was approved by the behavioral research ethics board of the University of British Columbia (H21-01800). The participants provided informed consent for the focus groups and were involved in the further design and development of the e-learning platform. All data were deidentified upon data collection. Individuals were renumerated for their participation via e-transfer. Participants each received $50 CAD (US $36.83) for their participation in a focus group, and participants were given $25 CAD (US $18.42) for each module storyboard that they reviewed.

## Results

### Needs Analysis

#### Previous Research

Dineen et al [10] demonstrated that coaches had little knowledge about MI and SSBC program content prior to taking the in-person training. Coaches also reported high levels of satisfaction with the in-person training topics.

#### SSBC Research Team Meetings

The SSBC research team made the following decisions based on 3 meetings. SSBC coaches needed to have basic knowledge about T2D prevention, specific SSBC session content (ie, Canada’s physical activity guidelines, daily added sugar limits, carbohydrates, and the talk test), what MI is, the spirit of MI, MI skills (ie, open-ended questions, affirmations, reflections, summaries and ask-tell-ask), the 4 processes of MI, how to deliver SSBC content using MI, and how to deliver culturally safe and inclusive care. Coaches would need to demonstrate skills associated with delivering the SSBC program to clients, which includes appropriately delivering the correct information using an MI-informed approach.

Once a coach completed the e-learning modules, the coach would demonstrate their skills through a mock session, providing an opportunity for assessment and provision of feedback. Sufficient knowledge would be determined by scoring a minimum of 70% on a knowledge test on T2D prevention, SSBC-specific content, and MI at the end of the training. The research team would develop the knowledge tests, drawing from content directly covered in the SSBC coach e-learning training. Finally, the coaches’ skills would be monitored and assessed by coding a random selection of audio recordings from sessions with clients using session-content checklists and the Motivational Interviewing Competency Assessment tool [14].

#### Focus Groups With SSBC Coaches

Invitation emails were sent to 15 SSBC coaches to participate in a focus group, including information about the purpose of the focus group and remuneration of time. A total of 9 SSBC coaches volunteered to take part in a focus group, and they were split into 3 equal-numbered focus groups based on their availability. See Table 1 for focus group participants’ demographics and experience as a coach. On average, each focus group was 62 minutes.

The results from the conventional content analysis showed that coaches wanted more information on the evoking process of MI, on transitioning from sustain talk to change talk, and an understanding of the effectiveness of MI. The coaches spoke about the importance of knowledge checks throughout the e-learning training, a collection of resources and commonly used documents (ie, a resource center), role-play videos demonstrating the skills being taught, and live sessions with a workshop facilitator to practice skills and receive feedback. The coaches suggested that modules be short in duration and for training to span over 2 weeks. The coaches’ recommendations also included the integration of a variety of learning methods through the use of didactic and user-engaging components. Additional resources suggested by coaches included video examples of delivering SSBC content using MI and various SSBC program delivery styles and scenarios. The participants desired a blended approach of asynchronous and synchronous learning, with an opportunity for practice and feedback.

**Table 1:** Focus group participants’ demographics and experience (N=9).

Individual-level factor		Values, n (%)
**Age (years)**		
	18-24	3 (33)
	25-34	3 (33)
	35-44	1 (11)
	45-54	2 (22)
**Gender identity**		
	Woman	8 (89)
	Man	1 (11)
**Race**		
	White	8 (89)
	White and Indigenous	1 (11)
**Education**		
	University certificate, diploma, or degree	4 (44)
	Apprenticeships, trades services, or diploma	1 (11)
	College, college d’enseignement general et professionnel, or other nonuniversity certificate or diploma	1 (11)
	High school	3 (33)
**Years of experience working in customer service**		
	1-2	1 (11)
	Greater than 5	8 (89)

### Design and Development

**Figure 1:**
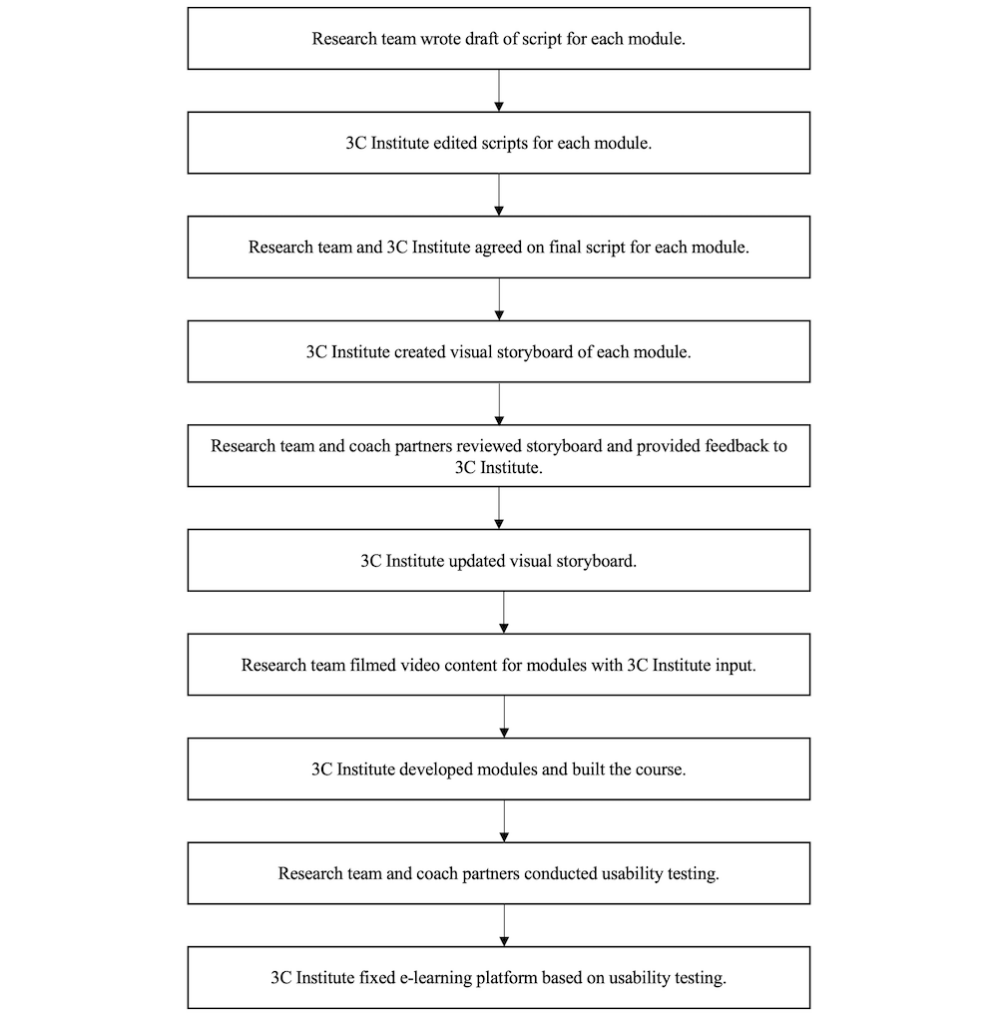
e-Learning platform design and development process with each group’s contributions.

The research team worked with 3C Institute and SSBC coaches over a series of 13 months (21 meetings) to design and develop the e-learning platform. Correspondence with the involved SSBC coaches to collect feedback was done over email. The flow of the entire design and development phases can be seen in Figure 1, showing each group’s contributions to development.

Information collected in the needs analysis stage was used to develop a blueprint for the e-learning platform, which was then reviewed by the 3C Institute. After consultations with 3C Institute and SSBC coaches, it was determined that the e-learning training would include seven modules—1 introducing SSBC and inclusivity, 1 covering SSBC-specific content, 4 covering MI, and 1 putting everything together. Additionally, 3C Institute provided suggestions for learning activities (eg, narrated videos, demonstration videos, and user-engaging activities) based on their expertise in developing e-learning platforms. All modules were designed to be asynchronous so coaches could move through them at their own pace, with user-engaging components and knowledge checks throughout the modules, and at the end of each module. The e-learning platform includes a resource center for coaches to access resources (eg, PDFs, video examples, and SSBC session paperwork).

The research team drafted 1 module script at a time. Scripts included what the narrator would say, and the content for the user-engaging activities and knowledge. The script for each module underwent numerous editing cycles between the 3C Institute and the research team before being sent to the 3C Institute multimedia artist. The multimedia artist developed storyboard proofs for each module. Storyboard proofs included still images representing what would be on the screen with the associated script. Feedback was collected from both the research team and SSBC coaches from the focus groups. For each module storyboard proof, 2 to 4 coaches provided feedback (dependent on their schedules and availability) and were remunerated for their time. After the storyboard proofs were approved by the SSBC research team and coaches, the narrators for the videos (first and last author) worked with a University of British Columbia Studios Okanagan producer and the 3C Institute editor to film the 7 modules. Talent was hired to act as coaches and clients in the role-play videos for the modules and the resource center. After each module was filmed, the 3C Institute multimedia artist and video director worked to edit and finalize the modules. An overview of the SSBC e-learning training platform is provided in Table 2 and a selection of screenshots from the online training platform are provided in Multimedia Appendix 1.

Based on decisions made following the needs analysis, the entire training process for new SSBC coaches includes 3 stages. First, the coaches must complete the 7 modules. Next, the coaches are required to schedule a 1-hour mock session with an SSBC research team member and are then provided with written feedback within 2 weeks of completing the mock session. Following the mock session, coaches are given access to the final knowledge test. After passing the final knowledge check, coaches are certified to take on SSBC clients independently.

Overall, the authors spent a total of 192 hours on this project (eg, preparing for and leading SSBC research team meetings and focus groups, analyzing data from needs analysis, designing blueprints, writing and editing scripts, developing knowledge check questions, emails, correspondence with SSBC coaches and talent, meetings with 3C Institute, filming, and usability testing). The final cost of developing the SSBC coach e-learning platform was ~US $80,000 (cost to hire 3C Institute and professional talent for filming).

**Table 2:** Overview of SSBC^a^ e-learning platform.

e-Learning component	Topics covered	Learning objectives	Module features
Module 1	Introduction to SSBC, prediabetes and T2D^b^, cultural safety, and inclusivity	Broadly describe T2D and prediabetes Explain how diet and exercise changes can help prevent T2D Describe what the SSBC program includes Understand why having an inclusive mindset is integral to being a SSBC coach Understand the need to learn and act with empathy when working with clients, regardless of their background and life experiences	Narrated video, role-play videos, and knowledge check questions
Module 2	SSBC session content, taking client measurements, tools associated with the program (eg, health tracking mobile app), and exercise protocols	Describe the content that comprises each of the 6 SSBC counseling sessions Understand the program tools and session documentation requirements Know the difference between SSBC’s moderate-intensity continuous training and high-intensity interval training exercise sessions and the weekly progressions for each Know which measurements to perform on your clients and when to take them	Narrated videos, demonstration videos, and knowledge check questions
Module 3	Introduction to MI^c^, definition of MI, and spirit of MI	Define MI and identify where it falls on the spectrum of counseling styles Describe each of the 4 elements of the spirit of MI Understand how to implement the spirit of MI in SSBC sessions	Narrated videos, role-play videos, and knowledge check questions
Module 4	MI skills: open-ended questions, affirmations, reflections, summaries (OARS^d^), and ask-tell-ask	Define and use the OARS skills Describe and use the method “ask-tell-ask” to provide information or advice to your clients	Narrated videos, role-play videos, and knowledge check questions
Module 5	Listening and responding to change talk, sustain talk, and ambivalence	Define change talk and sustain talk Define ambivalence Describe preparatory change talk and mobilizing change talk Understand which OARS skill will work best when a client engages in change talk, sustain talk, or ambivalence	Narrated videos, role-play videos, and knowledge check questions
Module 6	4 processes of MI: engaging, focusing, evoking, and planning	Describe the 4 processes of MI Understand how to use them in sessions with your clients	Narrated videos, role-play videos, and knowledge check questions
Module 7	Putting it all together: how to deliver the SSBC content using MI	Describe the elements of an SSBC session Understand how to provide SSBC content in a way that embodies the spirit and skills of MI	Narrated videos, role-play videos, and knowledge check questions
Resource Centre	Additional information on topics covered in modules, sessions scripts, session checklists, and video role-plays	N/A^e^	PDFs and role-play videos

^a^SSBC: Small Steps for Big Changes.

^b^T2D: type 2 diabetes.

^c^MI: motivational interviewing.

^d^OARS: open-ended questions, affirmations, reflections, summaries.

^e^N/A: not applicable.

### Test Usability

Results from the initial usability tests conducted by the research team and the SSBC coach partners showed high levels of learnability, efficiency, memorability, and satisfaction within the provided feedback. Some errors were recorded including technical bugs, which were corrected by 3C Institute.

## Discussion

### Principal Results

This paper provides a clear and transparent outline for the needs analysis, design, development, and initial usability testing of the SSBC coach training e-learning platform. Importantly, this process demonstrates how an IKT approach can be used with the TEL evaluation framework. To our knowledge, this is one of the first e-learning platforms that has been developed using an IKT approach. Engaging end users through this process increases the chances that they will find the e-learning platform useful, acceptable, and appropriate [15,16].

The development and use of e-learning platforms for SSBC coaches will improve the viability of the program as new coaches will be able to take the training from any location and at a time that is convenient for them. This process can be used to inform other T2D prevention and health programs currently training coaches in person. e-Learning has become a popular education tool; however, there is still a lack of meaningful engagement with end users and research outlining the development process.

A preliminary study demonstrated high levels of satisfaction and gain in coach knowledge after completing this SSBC e-learning platform [17]. The next steps include implementing and evaluating the SSBC coach e-learning platform to examine the real-world applicability and effects of the platform using the TEL evaluation framework phases 4-7. This will include documenting key events during implementation, assessing new coaches’ experience and satisfaction with the e-learning platform (ie, user-friendliness and appropriateness of the platform), assessing learning outcomes (Kirkpatrick levels 2-4) through changes in knowledge, behavior, SSBC client outcomes, and finally, estimating the total costs to develop and implement the e-learning platform, as well as determining viability.

Beyond the use of SSBC, other programs should consider an IKT approach in developing e-learning platforms and transparently report the process. Once training programs have been developed and implemented, it is crucial that research teams monitor coaches’ knowledge, skills, and behaviors and report on the fidelity of their coaches [18].

### Limitations

Only a small number of previous SSBC coaches were involved in the design and development of this e-learning platform, and their preferences for content and delivery style might not be reflective of that of all coaches who will take this e-learning training. However, working with 3C Institute allowed us to incorporate their expertise in e-learning platforms, content, and learning styles. This study does not examine the effectiveness of this training, and at this point, we cannot be certain that this training will improve coaches’ knowledge, skills, and behaviors. Future research will look at these learning outcomes.

### Conclusions

This is the first paper to outline the needs assessment, design, and development of an e-learning platform for a T2D prevention program. One of the main limitations of the current T2D prevention programs is the limited reporting of coach training modes and training fidelity. This paper demonstrates how an e-learning platform can be designed and developed for T2D prevention program coaches using an IKT approach combined with the TEL evaluation framework. It is expected that this training will be acceptable and effective because of the methods and approaches used in this study.

## Appendix

Multimedia Appendix 1 Screenshots from the e-learning platform.

## Abbreviations

IKT: integrated knowledge translation
MI: motivational interviewing
SSBC: Small Steps for Big Changes
TEL: technology-enhanced learning
T2D: type 2 diabetes

## Data Availability

The data sets generated and analyzed during this study are not publicly available to protect the intellectual property of SSBC but are available from the corresponding author on reasonable request.
