# Supplementary figures and images for: Designing and Developing Online Training for Diabetes Prevention Program Coaches Using an Integrated Knowledge Translation Approach: Development and Usability Study
Source: JMIR Form Res. 2024 Jan 26;8:e50942. doi: 10.2196/50942 (PMC10858411; doi:10.2196/50942)

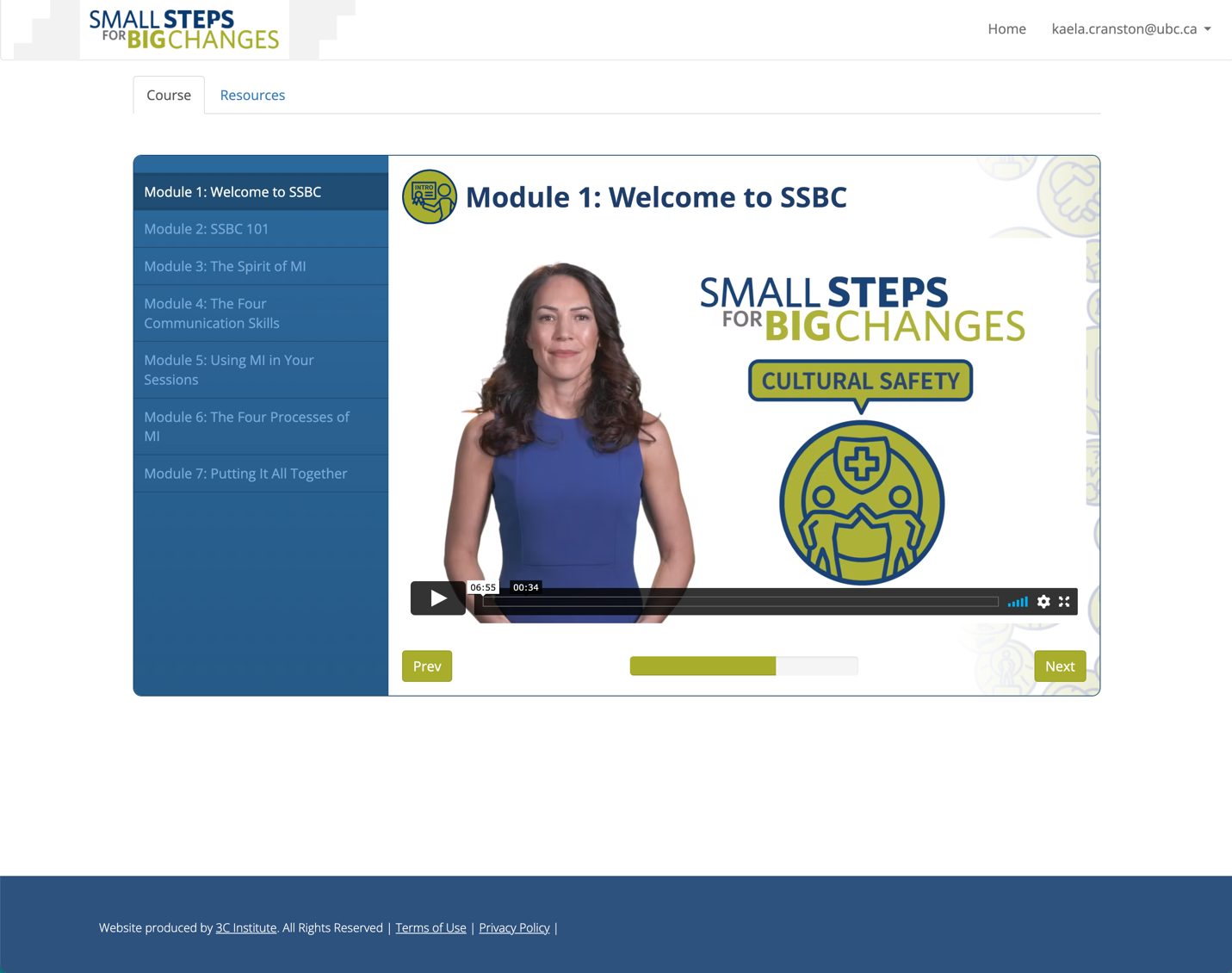


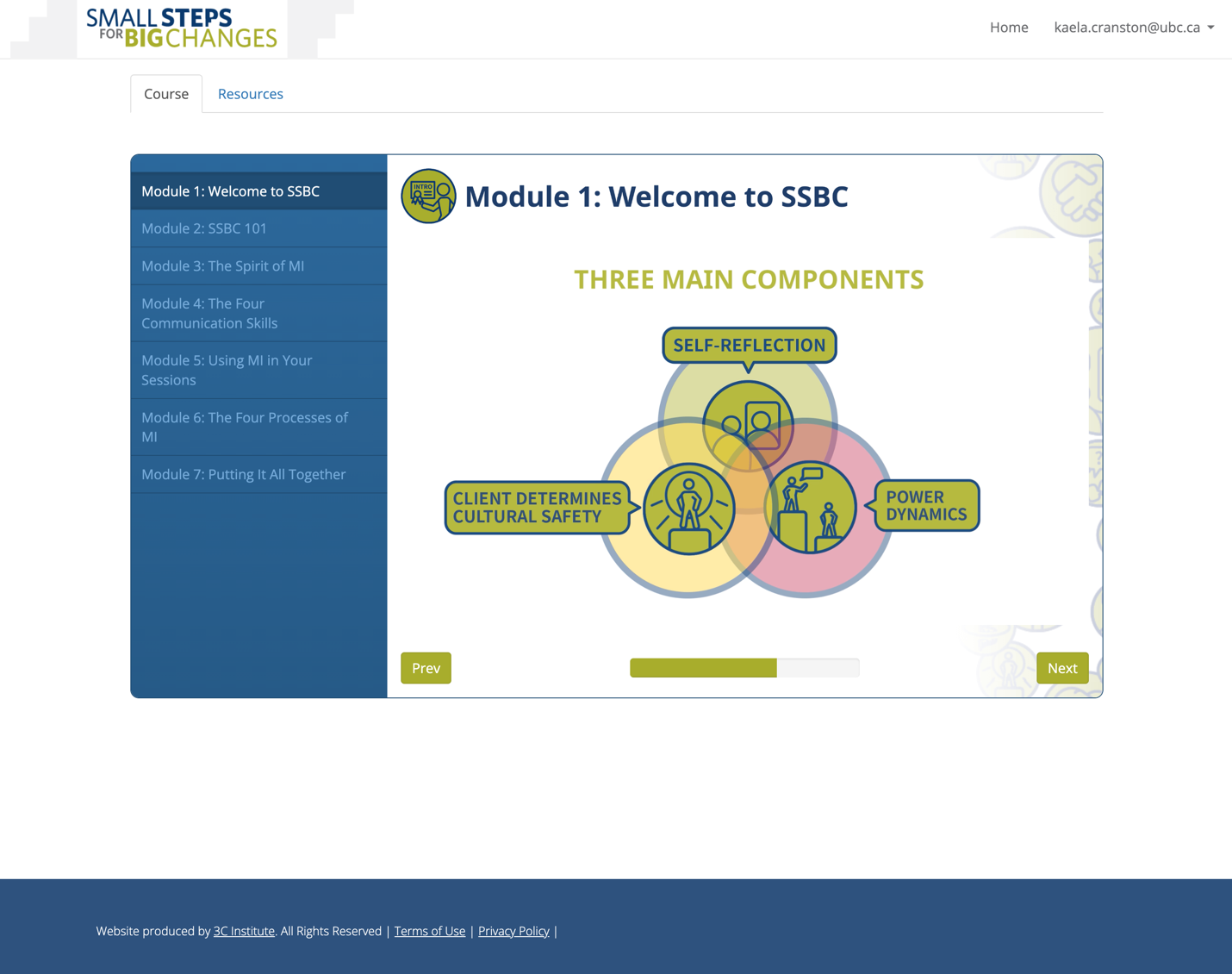


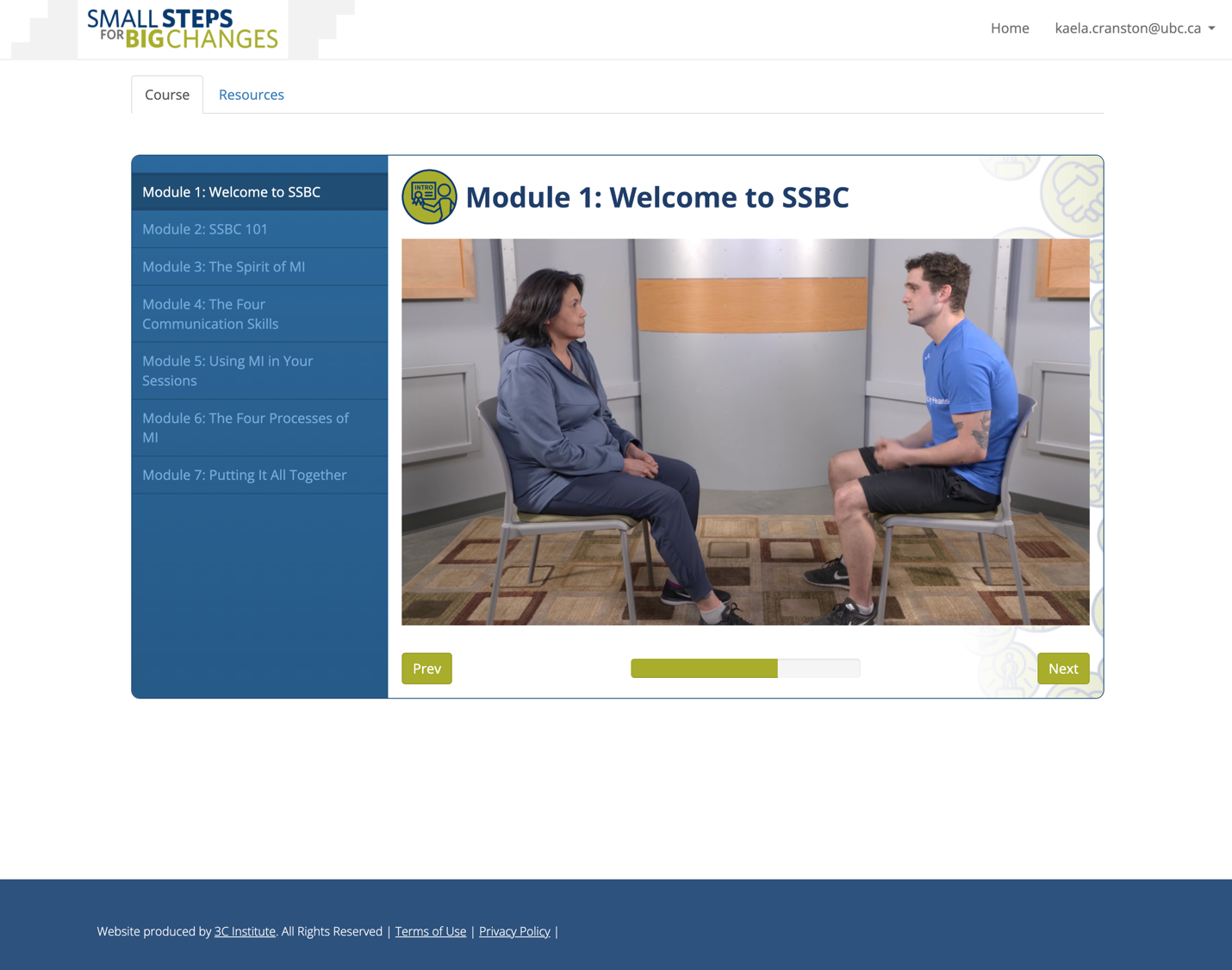


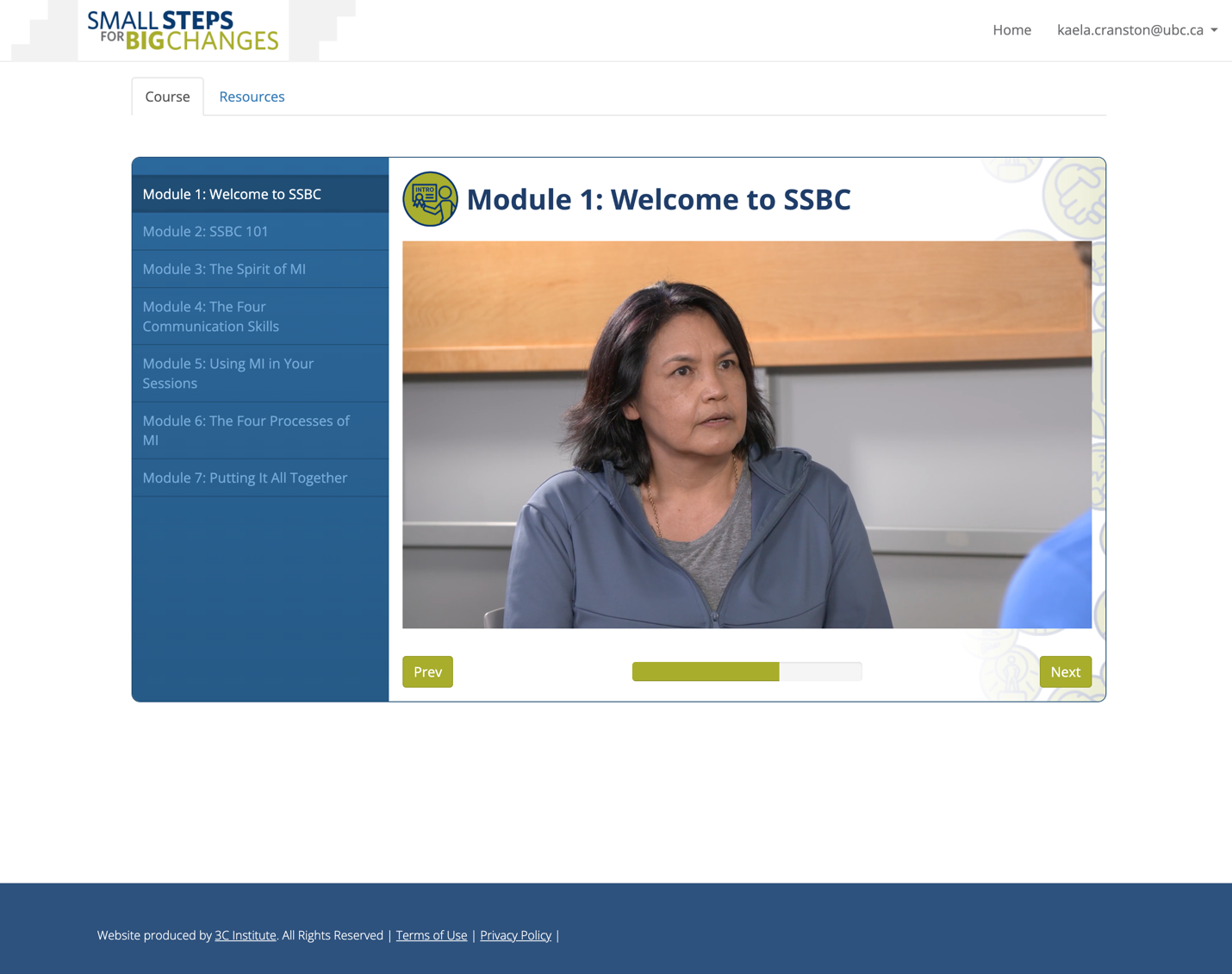


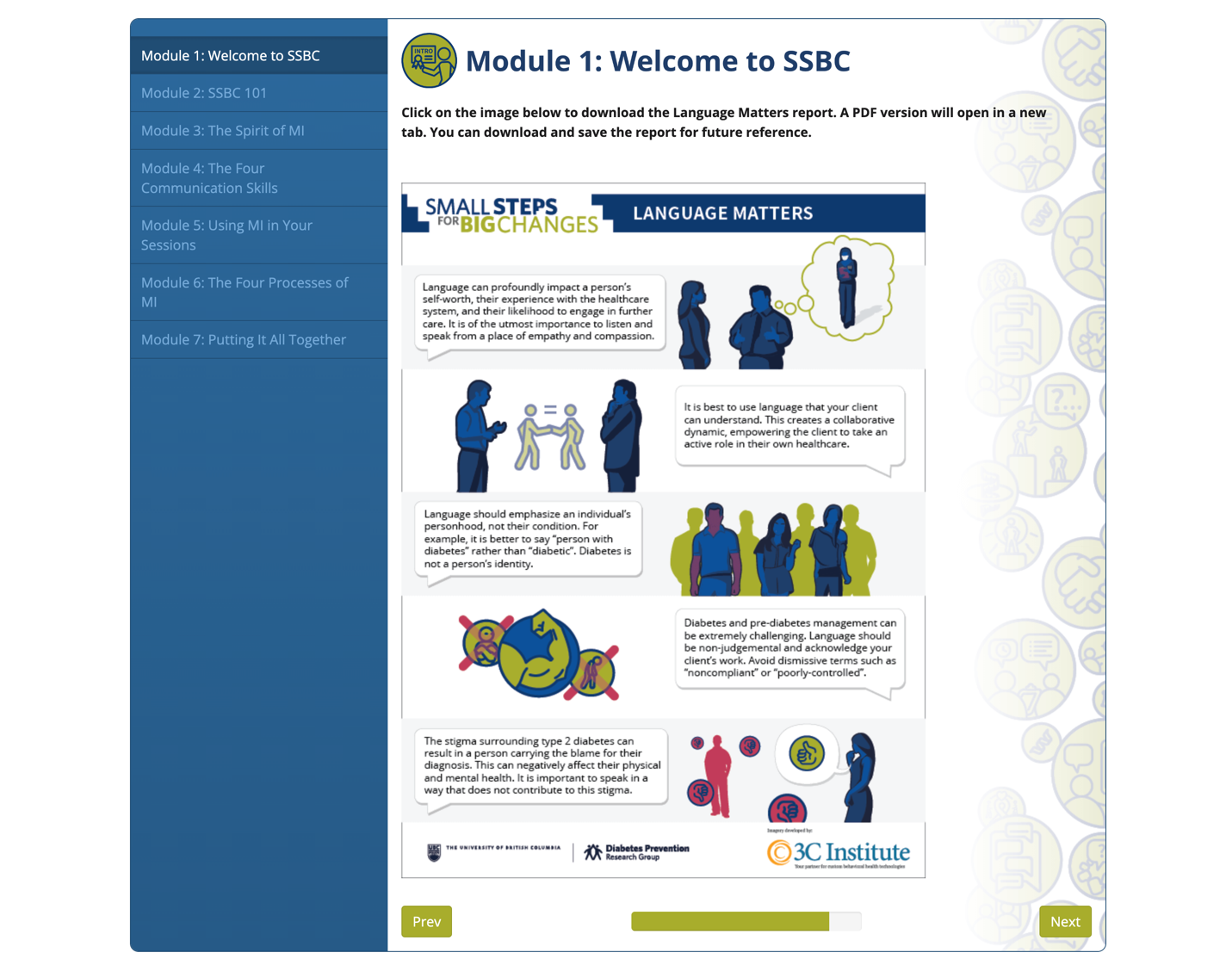


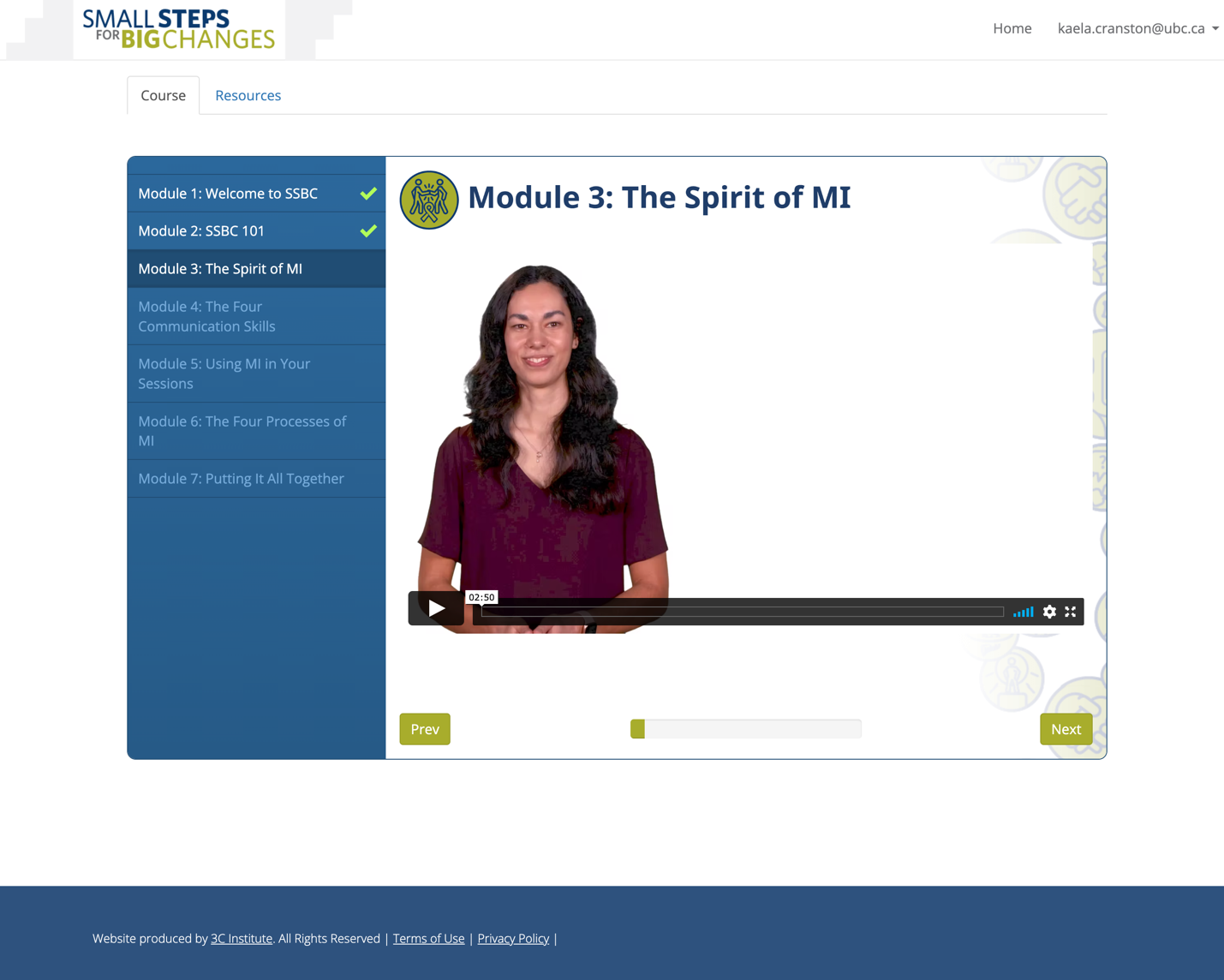


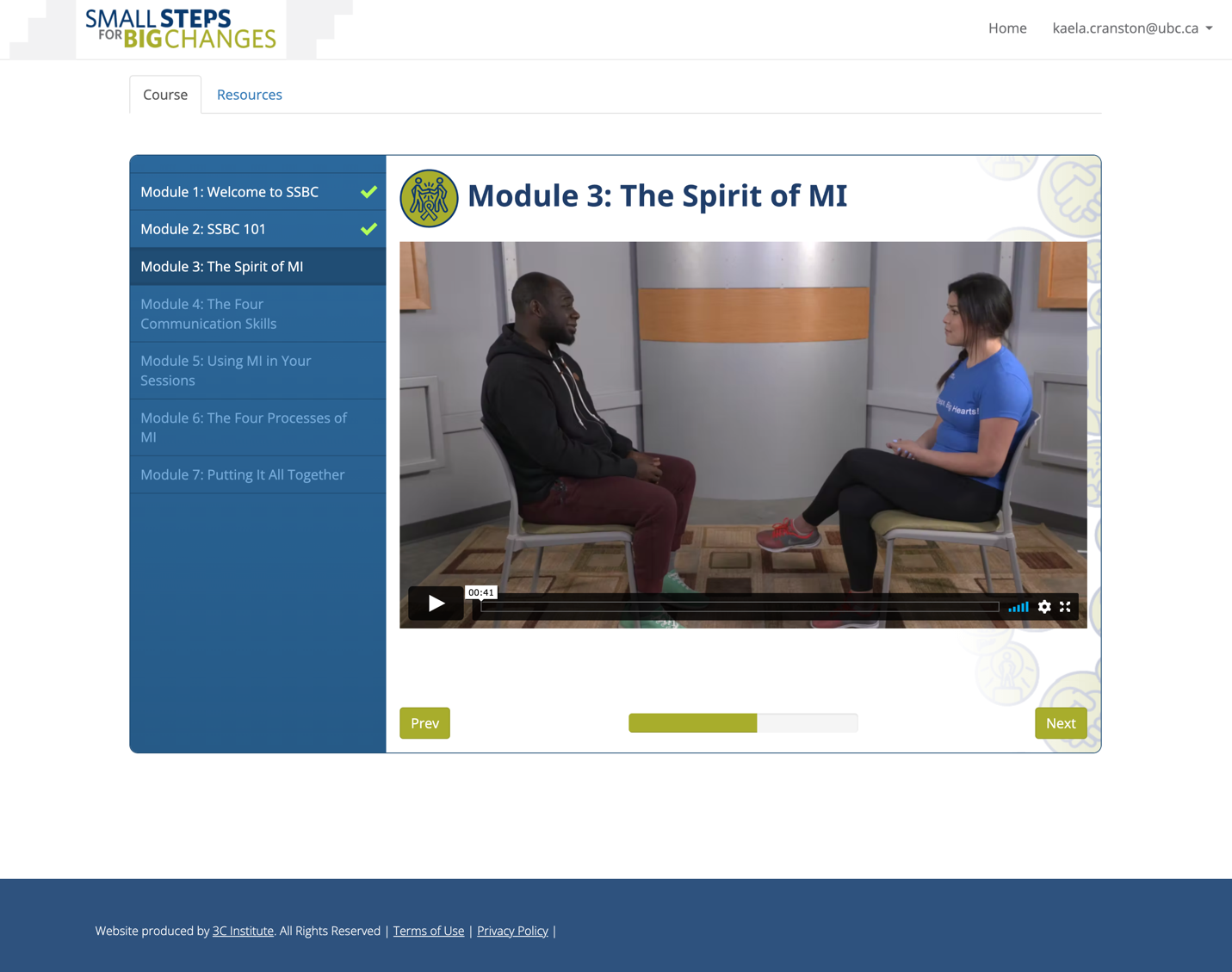


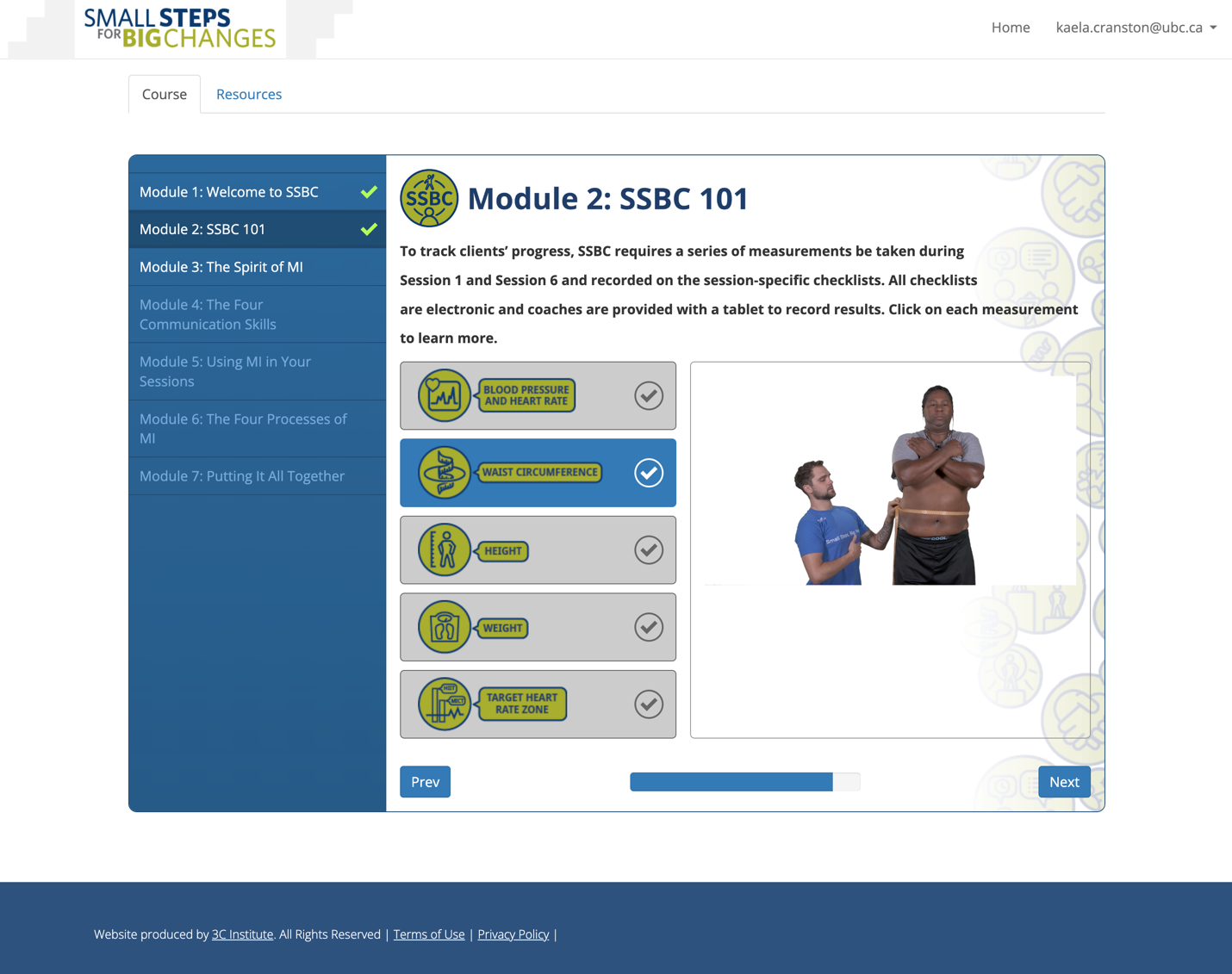


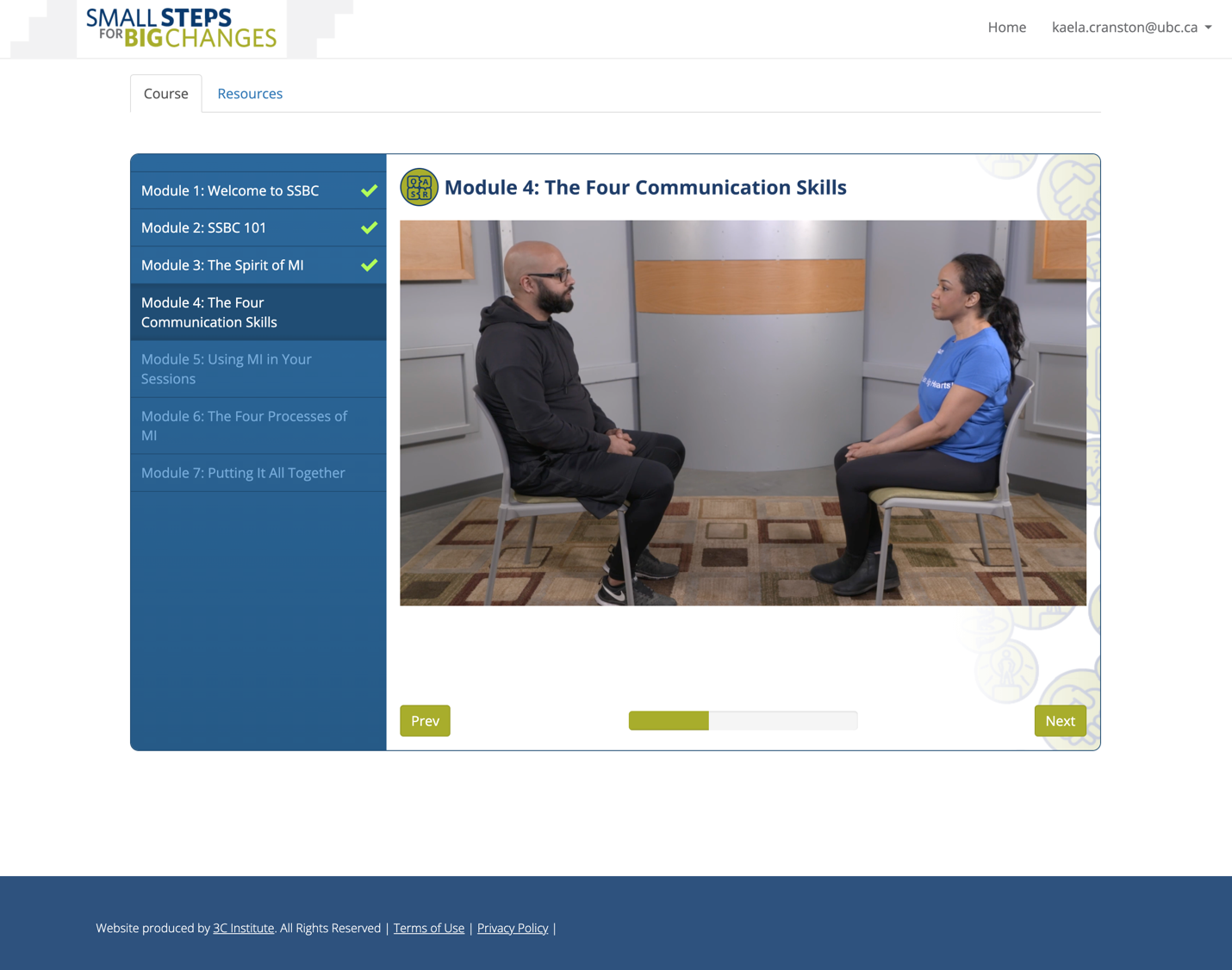

Supplement: Multimedia Appendix 1 [file formative_v8i1e50942_app1.docx]
